# Bioaccessibility of anthocyanins and bioactive compounds from Brazilian berries and their food matrix interaction: an *in vitro* gastrointestinal digestion study coupled to UHPLC‐ESI‐TQD‐MS/MS analysis

**DOI:** 10.1002/jsfa.70436

**Published:** 2026-01-29

**Authors:** Paulo Berni, Laís R Zandoná, Patrícia B Berilli, Livia Reguengo, Giulia Bonhin, Daniela CS Baldan, Mário R Maróstica

**Affiliations:** ^1^ Food Science and Nutrition Department School of Food Engineering, The State University of Campinas São Paulo Brazil; ^2^ Food Science Department School of Nutrition, Federal University of the State of Rio de Janeiro, UNIRIO Rio de Janeiro Brazil

**Keywords:** native fruits, INFOGEST, *Eugenia*, *Rubus*, anthocyanin metabolism, antioxidant potential, flavonoids

## Abstract

**BACKGROUND:**

Several commercial berries have been examined for anthocyanin composition, bioaccessibility, and bioactive effects, while Brazilian berries remain underexplored. This study aimed to access the effect of *in vitro* gastrointestinal digestion on anthocyanin bioaccessibility, stability, and antioxidant potential in black pitanga (*Eugenia uniflora* var. *rubra* Mattos), grumixama (*Eugenia brasiliensis*), nhamburi (*Rubus urticaefolius*), and barapiroca (*Eugenia involucrata*) – also called Rio Grande cherry – and explore its link with the food matrix composition.

**METHODS:**

The INFOGEST *in vitro* digestion model was applied to these berries, and anthocyanin were quantified using ultra‐high‐performance liquid chromatography–electrospray ionization–triple‐quadrupole–tandem mass spectrometry. Antioxidant potential, polyphenols, flavonoids, anthocyanins, and proximate composition were analyzed by conventional protocols. The results were used in performing analysis of variance, Pearson's R correlations, and multivariate principal component analysis to explore the food matrix key roles.

**RESULTS:**

Brazilian native berries, particularly black pitanga, nhamburi, and grumixama, were found to be rich sources of anthocyanins. Stability during *in vitro* digestion oscillated between berries and anthocyanin type, besides remaining over 40%. Bioaccessibility highlights are the cyanindin‐3‐glucoside and malvidin‐3,5‐diglucoside, which had the highest bioaccessibilities (between 45% and 68%). Black pitanga and nhamburi were able to provide the highest amounts of bioaccessible anthocyanins (477 and 1172 mg g^−1^, respectively). Black pitanga presented the highest ferric reducing antioxidant power and oxygen radical absorbance capacity in bioaccessible fractions. Multivariate statistics showed a clear correlation between centesimal composition and anthocyanin stability.

**CONCLUSION:**

The findings reveal complex interactions between food matrices, anthocyanin stability and bioaccessibility, and effective antioxidant potential for human health. This research emphasizes the use of Brazilian berries as good anthocyanin sources, highlighting black pitanga and nhamburi, while fostering consumer health and conservation of natural resources. © 2026 The Author(s). *Journal of the Science of Food and Agriculture* published by John Wiley & Sons Ltd on behalf of Society of Chemical Industry.

## INTRODUCTION

Consumption of fruit benefits health due to its nutritional components. Red‐to‐black fruits like berries are rich in bioactive compounds and dietary fiber, which help prevent chronic non‐communicable diseases (NCDs) such as metabolic syndrome and neurodegenerative disorders. These benefits are mainly due to polyphenols, especially flavonoids like anthocyanins. Anthocyanins, which give berries their blue, purple, red, or black color, have antimicrobial, antioxidant, anti‐inflammatory, and antimutagenic properties. They aid in managing NCDs such as obesity, type 2 diabetes, hypercholesterolemia, cancer, and cardiovascular diseases.^1^, ^2^, ^3^, ^4^, ^5^

Anthocyanins are included in the human diet mainly through the intake of berries and other red fruits. However, for anthocyanins to exert their benefits on the organism they must be bioaccessible to the body. Bioaccessibility (BA) refers to the amount of a compound that is released into the gastrointestinal tract and is available for absorption. For absorption to occur, these compounds must undergo digestive transformation and be soluble in the aqueous phase of digesta in order to come into contact with the intestinal epithelium.^6^

The extent to which anthocyanins become bioaccessible depends on several factors, such as the food matrix, the chemical nature of the compound, their localization within the food, structural properties, solubility, and stability throughout gastrointestinal digestion, with the food matrix possibly being the most critical. Studies indicate that plant cell walls, when broken during digestion, can interact with polyphenols, affecting their BA, which is contingent upon polyphenol molecular structure.^7^, ^8^, ^9^ Proteins within the food matrix can also influence polyphenols, either enhancing or reducing their BA.^10^ However, there remains a gap in understanding how diverse food matrices impact anthocyanin BA. Therefore, comprehending the changes occurring during digestion is crucial for estimating the BA and bioactivity of anthocyanins from various berries.

*In vitro* simulated digestion mimics human digestion processes, yielding relevant bioaccessible fractions of food at the intestinal level. These methods are fast, safe, reliable, and reproducible, without ethical restrictions like *in vivo* models. Victoria‐Campos *et al*.^11^ investigated purified extracts of anthocyanin BA using *in vitro* digestion, assessing stability, BA, and metabolism. Similarly, Scrob *et al*.^12^ evaluated the impact of gastrointestinal digestion on antioxidant activity of anthocyanins and ascorbic acid in lingonberry jams via *in vitro* digestion. Results indicate that the food matrix significantly affected the stability of bioactive compounds during digestion.

Commercial berries, typically grown in temperate climates, struggle to adapt to the Brazilian and tropical climates, leading to limited availability and high commercialization costs globally. Brazil boasts immense biodiversity, particularly in the Atlantic Forest and Cerrado biomes, harboring numerous unknown fruit species. Moreover, extensive deforestation and neglect of nature conservation endanger many native Brazilian fruit species. Nonetheless, researching these native fruits aligns with the United Nations Sustainable Development Goals, addressing social, economic, and environmental aspects to preserve resources, protect ecosystems, and promote long‐term well‐being for humans and the planet.^7^, ^13^

Among berries native from Brazil there are nhamburi (*Rubus urticaefolius*), black pitanga (*Eugenia uniflora* var. *rubra* Mattos), grumixama (*Eugenia brasiliensis*), and barapiroca (*Eugenia involucrata*) – also called cereja do Rio Grande. We decided to retain the indigenous names of these fruits, since this is crucial for preserving the ethnicity and traditional knowledge of the original Brazilian culture.^14^, ^15^ These berries are expected to have considerable concentrations of anthocyanins in their composition due to their purple‐black colors, and represent good alternatives to expensive overseas berries.

Previous studies have partially analyzed the therapeutic potential and chemical composition of these berries, showing promising results.^16^, ^17^, ^18^ Grumixama's anthocyanins mainly comprise delphinidin 3‐glucoside, cyanidin 3‐glucoside, and the aglycones delphinidin and cyanidin. Pitanga's ethanolic extract showed a preventive effect on metabolic parameters in rats fed a highly palatable diet, with cyanidin 3‐glucoside identified as the main anthocyanin. Ethanolic extracts from barapiroca exhibited promising phenolic composition, and *in vitro* antioxidant and antitumor potential, with cyanidin, delphinidin, and pelargonidin derivatives as the main anthocyanins.^16^, ^17^, ^18^ Although comprehensive studies on Brazilian berry composition are limited, pitanga, grumixama, nhamburi, and barapiroca also provide other nutrients and antioxidants, such as vitamin C, tannins, tocopherols, carotenoids, and minerals. Pitanga has a rich variety of carotenoids, especially lycopene, hydrolysable tannins, and vitamin C.^13^ Grumixama peel analysis found abundant condensed tannins and tocopherols.^13^, ^19^ Barapiroca supplies vitamins B – mainly riboflavin – tannins, provitamin A carotenoids, and tocopherols.^13^, ^19^ To the best of our knowledge, this is the first time that the proximal composition, anthocyanin profile, and antioxidant effects of nhamburi (*Rubus urticaefolius*) have been analyzed.

Beyond the promising results regarding the bioactivity and bioactive compounds of black pitanga, grumixama, nhamburi, and barapiroca berries, their anthocyanin behavior in gastrointestinal digestion still needs to be studied. Therefore, *in vitro* digestion studies that analyze anthocyanin BA and bioactivity are initial routes in elucidating the main mechanisms of action and highlight these berries as health promoters, while understanding and preserving Brazilian biodiversity. The aim of this work was to evaluate the effect of *in vitro* gastrointestinal digestion of black pitanga, grumixama, nhamburi, and barapiroca over anthocyanin BA and bioactivity, and correlate the results with the food matrix composition; its originality is based on the innovative and sustainability character of scientifically exploring these native Brazilian berries.

## MATERIAL AND METHODS

### Sample treatment and experimental design

Black pitanga (*Eugenia uniflora* var. *rubra* Mattos), grumixama (*Eugenia brasiliensis*), nhamburi (*Rubus urticifolius*) and barapiroca (*Eugenia involucrata*) were harvested at their maximum purple‐black coloration from orchards in the cities of Ribeirão Preto and Campinas, São Paulo, Brazil, and taken fresh to the laboratory. Initially, the fruits were washed in potable water and then their seeds and leaves were removed, leaving only the edible parts (pulp and skin), except for nhamburi, which have small and edible seed which were therefore preserved. The samples were homogenized for 1 min in Ultra‐Turrax (14 000 rpm, IKA, Staufen, Germany) and stored in polypropylene tubes at −18 °C until analysis.

**Figure 1:**
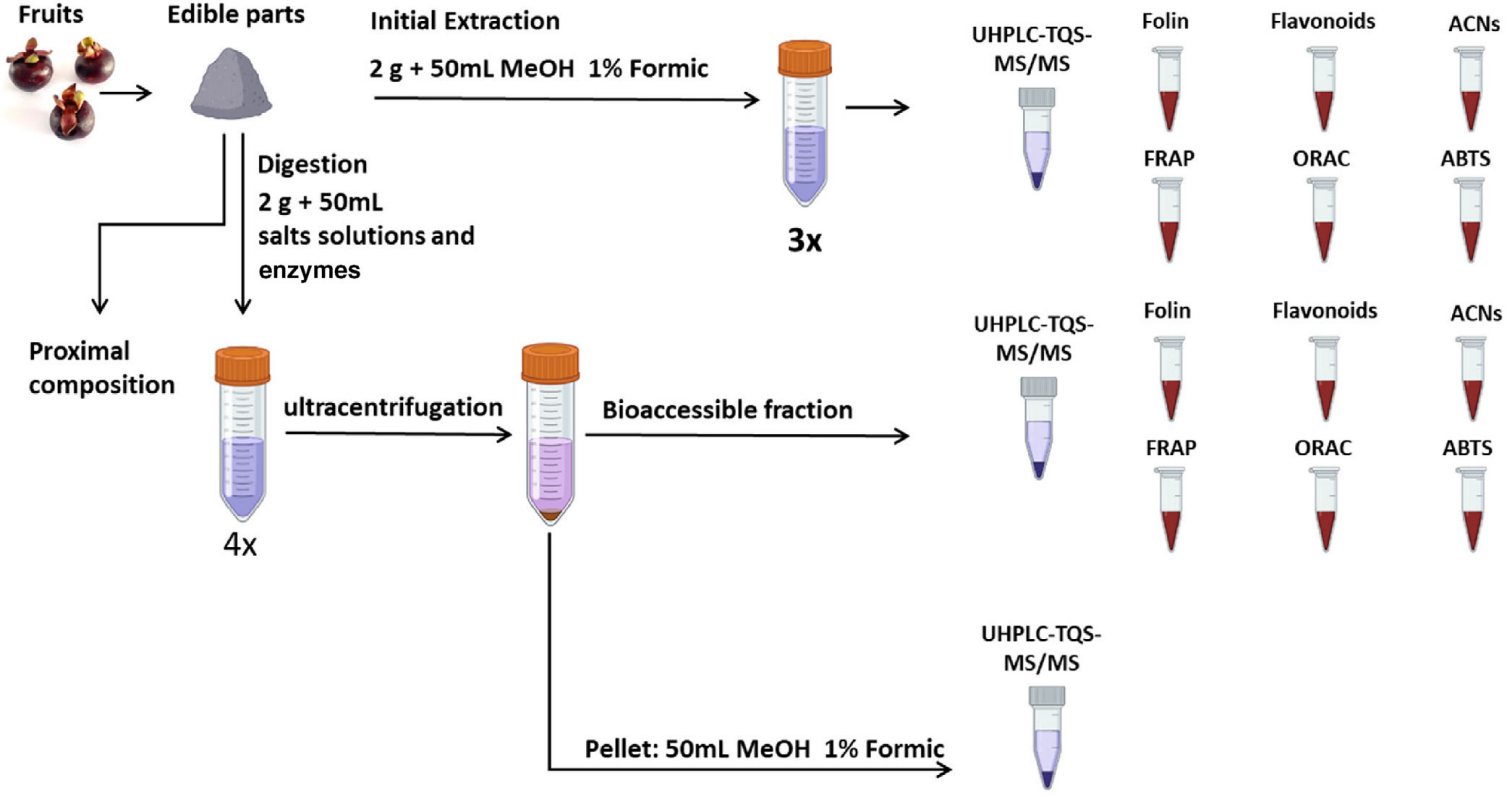
Experimental design.

The fruits were initially extracted as follows (Fig. 1): 2 g of homogenized samples was extracted with 15 mL of 80% methanol with 1% formic acid by successive maceration (three times) in glass flasks, placed in an ultrasonic bath for 10 min. Extracts were centrifuged at 5000 rpm in 15 mL polypropylene tubes for 5 min, collecting only the supernatant. The final volume after complete extraction was 50 mL, which was the same proportion of sample mass per volume in the *in vitro* digestion. These initial fruit extracts were filtered (0.22 μm) and analyzed by spectrophotometric analysis and ultra‐high‐performance liquid chromatography–electrospray ionization–triple‐quadrupole–tandem mass spectrometry (UHPLC‐ESI‐TQD‐MS/MS).

The fruits that were digested *in vitro* were analyzed as follows (Fig. 1): (i) the bioaccessible fraction – that is, the supernatant of digested samples after ultracentrifugation – was filtered (0.22 μm) for spectrophotometric analysis; (ii) aliquots of this bioaccessible fraction were also purified using SPE cartridges for UHPLC‐ESI‐TQD‐MS/MS analysis; and (iii) the pellets after ultracentrifugation (i.e., undigested residues) were extracted with solvent, as described above. The detailed *in vitro* digestion protocol is given below, as well details of the ultracentrifugation and purification steps.

### *In vitro* gastrointestinal digestion

The simulated *in vitro* digestion procedure used in this study followed strictly the parameters presented in the INFOGEST protocol.^20^ The only adaptation made in this protocol was the inclusion of uric acid (0.3 mg mL^−1^), urea (4 mg mL^−1^), and mucin (1 mg mL^−1^)^21^, ^22^ in the simulated salivary fluid (SSF). This adaptation was chosen because anthocyanins are visibly adherent to saliva and oral mucosa when anthocyanin‐rich fruits are ingested by humans.

Each sample was represented by four replications, with the first replicate used only for measuring the pH adjustment. Samples (2 g) were digested starting with the oral digestion process, including SSF with uric acid, urea, mucin, and α‐amylase. At each stage of digestion, the tubes were incubated in a water bath with orbital agitation (Dubnoff, ensuring temperature control and mixing throughout the digestion phase. After that, the tubes with the sample received a nitrogen blanket and were sealed with parafilm before incubation. For the *in vitro* digestion α‐amylase (75 U mL^−1^), pepsin (2000 U mL^−1^), pancreatin (100 U mL^−1^), bile (10 mmol L^−1^), lipase (60 U mL^−1^), and electrolyte solutions were used following the protocol, as well as the pH values and incubation parameters – that is, oral phase pH 7 and 2 min at 37 °C, gastric phase pH 3 and 2 h at 37 °C, and intestinal phase pH 7 and 2 h at 37 °C.

After *in vitro* digestion, the tubes were removed from the water bath and placed on ice, then samples were immediately acidified with 0.5 mL formic acid for maintaining anthocyanin stability. The tubes were then submitted to an ultracentrifugation step (24 000 × *g* at 4 °C for 45 min) to separate the bioaccessible fraction, which was the supernatant containing the released and soluble compounds (Fig. 1). With the help of a syringe and needle, as much as possible of the bioaccessible fraction was collected. This fraction was then filtered using 0.22 μm cellulose filters, passed with nitrogen, sealed with parafilm and frozen at −80 °C. The retained pellets were extracted as described above, representing the nondigested residue, and used for calculating the stability of the anthocyanins.

The bioaccessible fractions obtained were cleaned from polar compounds such as sugars, salts, acids, and other interfering substances with SPE cartridges (Bond Elut C18, Agilent Technologies, Santa Clara, CA, USA) for injection in the UHPLC‐ESI‐TQD‐MS/MS system.^23^, ^24^ We performed preliminary tests injecting bioaccessible fractions with and without SPE cleaning of samples, finding that SPE‐clean analysis produced lower noise, better‐shape chromatographic peaks, and relative recovery higher than 85%. SPE cartridges were activated with 1 mL methanol with 1% formic acid. Next, 600 μL of the bioaccessible fraction was passed through the cartridge. Then, 2 mL acidified water (1% acid formic) was passed through the SPE, retaining the anthocyanins and discarding the waste. Then, 300 μL acidified methanol was added and the content was filtered and collected directly in the vials for injection into the UHPLC‐ESI‐TQD‐MS/MS system. BA and stability were calculated as previously reported,^21^, ^25^ according to the following equations (Equ 1 and 2):

(1) $$\mathrm{BA}\;\left(\%\right)=\left(\frac{\text{Bioaccessible fraction}}{\text{Initial extract}}\right)\;\times \;100$$

(2) $$\text{Stability}\;\left(\%\right)=\left(\frac{\text{Bioaccessible fraction}+\text{Pellet extract}}{\text{Initial extract}}\right)\;\times \;100$$

### UHPLC‐ESI‐TQD‐MS/MS

The identification and quantification of the anthocyanin profile were analyzed with a UHPLC‐ESI‐TQD‐MS/MS system (Waters Corp., Milford, MA, USA) equipped with a triple‐quadrupole mass detector with an electrospray ionization source (Xevo TQS Zspray, Waters). A validated rapid method for separation and quantification of the main anthocyanins was chosen.^26^ The samples were injected into the UHPLC‐ESI‐TQD‐MS/MS system, with the following conditions: 0.35 mL min^−1^, 1% formic acid (A) and acetonitrile with 1% formic acid (B) were used as mobile phase in a gradient starting at 1 min from 90:10 (A:B) to 50:50 (A:B) in 4 min; total runtime was 9 min; and the column was an ACQUITY UPLC BEH C18 1.7 μm (Waters). Direct infusion of standards was performed to determine the optimum identification parameters at the mass spectrometry module using the software's Intellistart function. The temperatures for the ionization source and desolvation were 150 and 300 °C, respectively. The voltages in the cone and capillary were 3.1 kV and 49 V, respectively. The mass spectrometer was operated in positive mode. Pure standards of cyanidin 3‐glucoside chloride, delphinidin 3‐glucoside chloride, pelargonidin 3‐glucoside chloride and malvidin 3,5‐diglucoside chloride (Phytolab GmbH & Co., Vestenbergsgreuth, DE), were used to construct four calibration curves with distinct scales of concentration. Information on the calibration curve is presented in Supporting Information, Data S1.

The exact mass determined for each standard and their respective fragments was: cyanidin 3‐glucoside, 449.107; 287.055/162.052; delphinidin 3‐glucoside, 465.380; 303 048/162.052; pelargonidin 3‐glucoside, 433.114; 271.060; and malvidin 3,5‐diglucoside, 655.481; 493.43/162.052. Since these parameters were consistent with the literature,^9^, ^16^, ^17^, ^18^, ^26^ additionally, other expected anthocyanins were searched using mass data from authors who analyzed *Eugenia uniflora*,^16^ *Eugenia brasiliensis*,^17^ and *Eugenia involucrata*.^18^ The mode scan MRM (multiple reaction monitoring) was applied to searching simultaneously for 15 different compounds. Therefore, we were able also to identify in the samples the anthocyanins malvidin 3‐arabinoside/xyloside, cyanidin 3‐arabinoside, malvidin 3‐glucoside, cyanidin 3‐xyloside, delphinidin 3‐arabinoside, delphinidin‐3‐xyloside, and petunidin 3‐glucoside/galactoside.

### Proximal composition

Proximate composition was determined following internationally recognized procedures standardized by the Adolfo Lutz Institute^27^ and AOAC,^28^ as detailed below.

Moisture content was determined by oven‐drying at 105 °C until constant weight, using a forced‐air drying oven, according to AOAC.^28^ Ash content was determined by incineration in a muffle furnace at 550 °C until complete mineralization, according to AOAC.^28^

Protein content was determined by the Kjeldahl method,^27^ which involves digestion with concentrated H_2_SO_4_ in the presence of catalyst, distillation of the released ammonia in a Kjeldahl distillation apparatus, and titration with standardized HCl solution. Total nitrogen values were converted to crude protein using a factor of 6.25, as recommended by FAO/WHO and Southgate.^27^

Lipid content was determined according to the Bligh and Dyer method,^27^ which involves homogenization of the sample with chloroform and methanol (2:1, v/v) using a high‐speed homogenizer, phase separation with water, and quantification of the lipid fraction after solvent evaporation under reduced pressure.

Dietary fiber (total, soluble, and insoluble) was determined by the enzymatic–gravimetric method using MES‐Tris buffer, according to Lee *et al*.^27^ Total carbohydrate content was calculated by difference, subtracting the sum of moisture, protein, lipid, ash, and dietary fiber from 100. The energy value (kcal) was estimated using Atwater conversion factors (4 kcal g^−1^ for protein and carbohydrate, and 9 kcal g^−1^ for lipid). All determinations were performed in triplicate, and results were expressed on a fresh weight basis.

### Spectrophotometric analysis

Total polyphenol compounds were determined using the Folin–Ciocâlteu reagent, as described by Swain and Hillis;^29^ the results were expressed as gallic acid equivalents (μg GAE). Total flavonoids were determined according to Zhishen *et al*.;^30^ the results were expressed as catechin equivalents (μg CE). The monomeric anthocyanins were quantified according to the pH‐differential method from Giusti and Wrolstad.^31^ The final absorbance was obtained using Eqn (1), and the anthocyanin content was calculated as cyanidin‐3‐*O*‐glucoside (C3G) using Equ (2):

(3) $$A=\left[\left(A_{520\;\mathrm{nm}}–A_{700\;\mathrm{nm}}\right)\;\mathrm{pH}\;1.0\right]–\left[\left(A_{520\;\mathrm{nm}}–A_{700\;\mathrm{nm}}\right)\;\mathrm{pH}\;4.5\right]$$

(4) $$C\;\left(\mathrm{mg}\;C3100G\;\;g^{-1}\right)=A.\mathrm{MW}.\mathrm{DF}/\xi .L$$

where *A* is absorbance, *C* is concentration, MW is molecular weight (449.2), DF is dilution factor, *ξ* is molar absorptivity (26.900 mol L^−1^), and *L* is path length (cm).

The oxygen radical absorbance capacity (ORAC) assay was carried out based on a method previously described by Ou *et al*.^32^ The areas under the fluorescence decay curves were used to calculate the results, which were expressed as Trolox equivalents (μmol TE). Ferric reducing antioxidant power (FRAP) was performed according to Benzie and Strain.^33^ Results were expressed as Trolox equivalents (μmol TE). The radical cation ABTS^•+^ was used to determine the antioxidant capacity according to the method described by Rufino *et al*.^34^ Trolox was used as a standard for the calibration curve and the results were expressed as Trolox equivalents (μmol TE). Absorbances/fluorescences were read on a Synergy HT microplate reader (BioTek, Winooski, VT, USA) coupled to the data software program Gen5 2.0.

### Statistics

Statistics included Student's *t*‐test and analysis of variance (ANOVA; one‐way and two‐way), followed by Tukey's test to compare two or multiple means. A value of *P* ≤ 0.05 was considered significant. Analysis was performed using MiniTab 17 (Minitab LLC, State College, PA, USA) and GraphPad Prism 8.4.3 (GraphPad LLC, Boston, MA, USA) software. Data were expressed as mean ± standard deviation. Further statistical analysis in Metaboanalyst 5.0 (https://www.metaboanalyst.ca/) were used to correlate the features among them and with the samples after *in vitro* gastrointestinal digestion. After normalization by sum, square root transformation, and auto scaling, proximal composition, compounds BA and stability, total compound content, and antioxidant capacity features were submitted to principal component analysis (PCA) and Pearson's R correlation with a cutoff of *P* > 0.7.

## RESULTS

### Identification and quantification of anthocyanins by UHPLC‐ESI‐TQD‐MS/MS

Identification and relative abundance of main anthocyanins in the fruits were obtained using UHPLC‐TQD‐MS/MS, and data are presented in Table 1. Exact identification of cyanidin 3‐glucoside, delphinidin 3‐glucoside, pelargonidin 3‐glucoside and malvidin 3,5‐diglucoside were confirmed through the injection of these standards, confirming that these compounds are present in all four berries analyzed. This identification showed that the compounds' mass, fragments, and parameters of identification were identical to those found in the literature.^16^, ^17^, ^18^, ^26^ Then, in addition to the four standards used, it was also possible to identify seven other derivatives of these compounds: malvidin 3‐arabinoside/xyloside; malvidin 3‐glucoside; cyanidin 3‐arabinoside; cyanidin 3‐xyloside; delphinidin 3‐arabinoside; delphinidin‐3‐xyloside; and petunidin 3‐glucoside/galactoside. Besides mass and fragmentation, elution order was also utilized for compound identification.

**Table 1:** UHPLC‐MS/MS identification and relative abundance (RA) of anthocyanin.

Anthocyanin identity	MS ion parent	MS/MS daughter fragment		RT*(min)	Black pitanga RA* (%)	Grumixama RA (%)	Nhamburi RA (%)	Barapiroca RA (%)
Delphinidin 3‐glucoside	465	303	162	1.6	0.31%	1.25%		5.23%
Malvidin 3‐arabinoside/xyloside	463	331	132	1.71	0.02%	0.02%	0.04%	
Malvidin 3,5‐diglucoside	655	493	162	1.97	0.02%	0.04%	0.03%	
Cyanidin 3‐glucoside	449	287	162	2.45	98.46%	97.29%	99.27%	90.05%
Malvidin 3‐glucoside	493	331	162	2.55	0.01%		0.02%	0.03%
Pelargonidin 3‐glucoside	433	271	162	3.64	0.82%	0.21%	0.50%	1.63%
Cyanidin 3‐xyloside	419	287	132	4	0.30%	1.09%	0.06%	0.88%
Delphinidin 3‐arabinoside	435	303	132	4.43	0.01%	0.001%	0.03%	0.67%
Delphinidin‐3‐xyloside	435	303	132	4.89	0.06%	0.11%	0.05%	1.50%
Petunidin 3‐glucoside/galactoside	479	317	162	5.04				0.01%

* RT, retention time; RA, Relative Abundance.

The major anthocyanin present in all berries is cyanidin 3‐glucoside, being more abundant in nhamburi (99.3%), followed by black pitanga (98.5%), grumixama (97.3%), and barapiroca (90%). Despite being less abundant than cyanidin 3‐glucoside, small fractions of cyanidin 3‐xyloside were found in all fruits: grumixama (1.09%); barapiroca (0.88%); black Pitanga (0.30%); and nhamburi (0.06%). Pelargonidin 3‐glucoside is also present in small quantities in all fruits, with relative abundance of 1.63% (barapiroca), 0.82% (black pitanga), 0.50% (nhamburi) and 0.21% (grumixama). Highlights for delphinidin 3‐glucoside and delphinidin 3‐xiloside were found in barapiroca, with relative abundances of 5.2% and 1.5%, respectively.

Table 2 presents the quantification of four anthocyanins in initial fresh berries and their quantities in bioaccessible fractions after *in vitro* digestion. As observed, cyanidin 3‐glucoside presented the highest concentration in all berries following the order: black pitanga (1156 μg g^−1^) > barapiroca (1002 μg g^−1^) > nhamburi (954 μg g^−1^) > grumixama (251 μg g^−1^). Black pitanga and grumixama did not present significant differences between quantities of delphinidin 3‐glucoside and malvidin 3,5‐diglucoside. Delphinidin 3‐glucoside presented the second highest concentration for barapiroca > nhamburi > grumixama; however, for black pitanga the second highest concentration was pelargonidin 3‐glucoside. The lower quantities of anthocyanins determined was malvidin 3,5‐diglucoside in all berries.

**Table 2:** UHPLC‐TQD‐MS/MS quantification of anthocyanins in the fresh berries and in their bioaccessible fractions after *in vitro* digestion

Anthocyanin	Black pitanga	Grumixama	Nhamburi	Barapiroca
	*Initial* (μg g^−1^ w/w)			
Delphinidin 3‐glucoside	53.03 ± 4.65c	52.83 ± 4.31c	155.68 ± 4.60b	173.87 ± 4.91a
Malvidin 3,5‐diglucoside	1.92 ± 0.63b	1.17 ± 0.21b	3.14 ± 0.06a	1.66 ± 0.01b
Cyanidin 3‐glucoside	1156.80 ± 45.7a	251.10 ± 21.7c	954.80 ± 85.4b	1002.4 ± 30.4b
Pelargonidin 3‐glucoside	68.40 ± 1.21b	38.89 ± 3.01d	51.42 ± 1.14c	94.12 ± 0.53a
	
	*Bioaccessible* (μg g^−1^ w/w)			
Delphinidin 3‐glucoside	17.46 ± 0.72bc	7.60 ± 0.10c	40.90 ± 1.70a	28.97 ± 8.75b
Malvidin 3,5‐diglucoside	0.82 ± 0.03b	0.82 ± 0.014b	1.87 ± 0.15a	0.00 ± 0.00c
Cyanidin 3‐glucoside	435.60 ± 11.80b	120.37 ± 9.21d	642.10 ± 30.4a	246.18 ± 6.50c
Pelargonidin 3‐glucoside	22.80 ± 0.20a	5.50 ± 0.20c	10.46 ± 0.35b	24.66 ± 2.84a

Values are expressed as mean ± SEM (*n* = 3). Different lower‐case letters in the same row indicate significant differences among fruits, according to Tukey's test (*P* < 0.05).

The quantities of anthocyanins determined in bioaccessible fractions after *in vitro* digestion are also present in Table 2. Despite the term 'bioaccessibility' being more frequent, the amount of bioactive that is released and is bioaccessible is much more relevant biologically. On this basis, cyanidin 3‐glucoside showed the highest amounts in bioaccessible fractions between the measured anthocyanins, whereas its bioaccessible concentration was higher for nhamburi (642 μg g^−1^), followed by black pitanga (435 μg g^−1^), barapiroca (246 μg g^−1^), and grumixama (120 μg g^−1^). Malvidin 3,5‐diglucoside was no longer found after *in vitro* digestion; however, this compound presented low amounts on initial berries, and was then probably below the detection limit. Delphinidin 3‐glucoside remained the second most present compound in the bioaccessible form for nhamburi > barapiroca > grumixama, while, for black pitanga, pelargonidin 3‐glicoside was the second most quantified compound in the bioaccessible fraction. According to the results, nhamburi is the berry that has higher amounts of bioaccessible delphinidin 3‐glucoside, malvidin 3,5‐diglucoside, and cyanidin 3‐glucoside.

### Stability and BA of anthocyanins analyzed by UHPLC‐ESI‐TQD‐MS/MS

**Figure 2:**
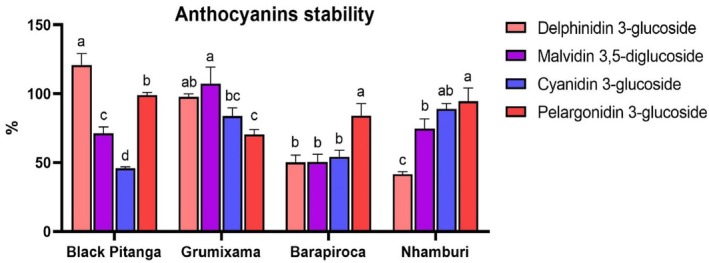
Stability after *in vitro* gastrointestinal digestion of individual anthocyanins analyzed by UHPLC‐TQD‐ESI‐MS/MS. ANOVA and Tukey tests were run separately for each fruit. Letters above bars indicate statistical differences (*P* < 0.001) between anthocyanins of each fruit analyzed.

Stability data (Fig. 2) represent the total amounts of anthocyanins recovered after *in vitro* digestion, including both the bioaccessible fraction and the undigested fraction, relative to the initial amounts, thereby reflecting the compounds' chemical stability throughout the digestive process. It represents the retention of anthocyanins – that is, the resistance to degradation during *in vitro* digestion steps. Moreover, stability helps to infer the anthocyanins that were chemically transformed during the digestion process, but may have biological relevance – that is, metabolized. The stability during *in vitro* digestion for all quantified anthocyanins and samples are above 40%. Delphinidin 3‐glucoside was the most stable compound for black pitanga (120.8%), followed by pelargonidin 3‐glucoside (99%). Malvidin 3,5‐diglucoside was the compound with the highest stability for grumixama (107.3%), while delphinidin 3‐glucoside presented similar stability (97.7%). The most stable compound for barapiroca and nhamburi was pelargonidin 3‐glucoside (84.1% and 94.6%, respectively). For barapiroca berry, malvidin 3,5‐diglucoside, delphinidin 3‐glucoside and cyanidin 3‐glucoside showed similar stability (around 50%). Delphinidin 3‐glucoside was the less stable compound for barapiroca and nhamburi (50.20% and 41.59%, respectively). Despite having a small bioaccessible amount, the compound malvidin 3,5‐diglucoside showed a stability above 50% for all berries analyzed. The finding of stabilities over 100% (Fig. 2) is not surprisingly since variability of stability results includes the error of three different steps of a biological process independently analyzed (initial, bioaccessible, and residual), and the final amounts of anthocyanins – that is, after *in vitro* digestion – are not statistically higher than the initial samples.

**Figure 3:**
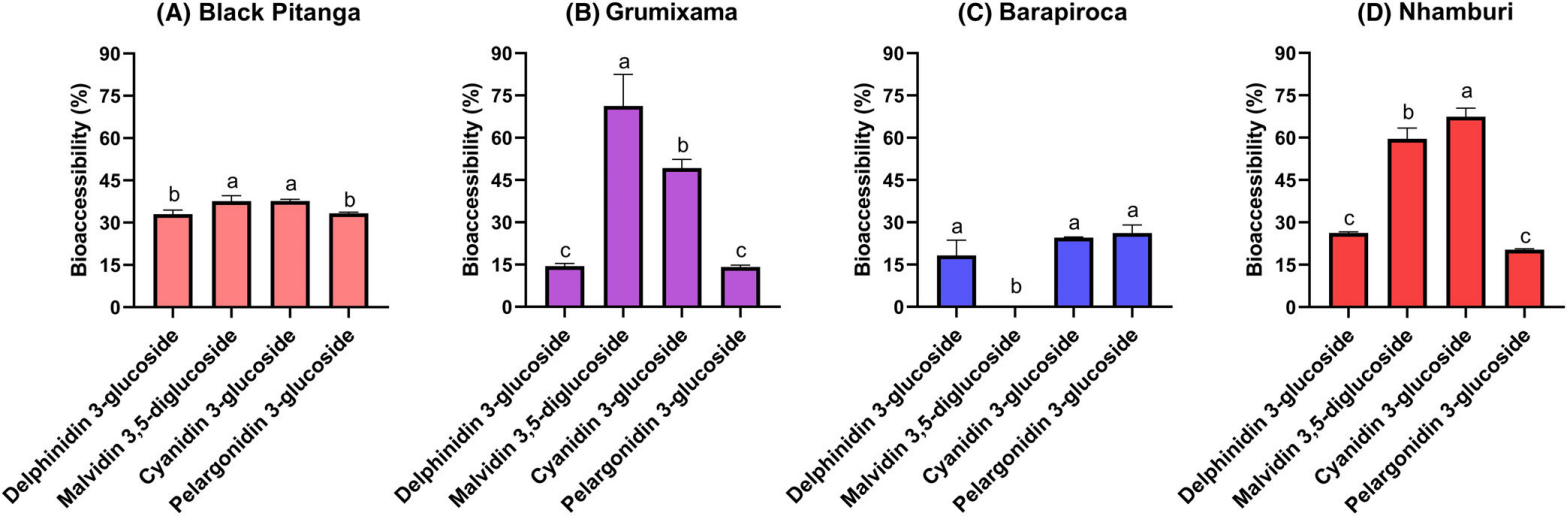
*In vitro* bioaccessibility of individual anthocyanins analyzed by UHPLC‐TQD‐ESI‐MS/MS. Letters above bars indicate statistical differences between anthocyanins of each fruit according to ANOVA and Tukey tests (*P* < 0.001).

BA data (Fig. 3) represent the fraction of each measured anthocyanin that is released from the food matrix during digestion, remains chemically stable, and is soluble in the aqueous fraction, thus being available for absorption in the gastrointestinal tract. These data, as a percentage, permits us to compare different samples regarding the efficiency with which a bioactive compound becomes bioaccessible. It also permits us to initiate explanations concerning mechanisms of action and BA modulation, like food matrix interference. Nhamburi presented the highest BA of cyanidin 3‐glucoside (67.4%), followed by grumixama (47.9%), black pitanga (37.6%), and barapiroca (24.5%). Black pitanga did not show significant differences between anthocyanin BA, staying at around 35%. Malvidin 3,5‐diglucoside was the most bioacessible compound in grumixama; nonetheless, this compound did not present relevant amounts in the berries. Barapiroca was the berry with the lowest BA for all anthocyanins, excluding pelargonidin 3‐glucoside at 20%, which was higher only than grumixama (14%). A limitation of these experiments is that the extraction and analytics chosen do not focus on bound or polymerized forms of anthocyanins, which could underrepresent its role in stability and BA results.

### Total phenolic compounds, total flavonoids, and total monomeric anthocyanins

Results from spectrophotometric analyses of the bioactive compounds before and after simulated digestion of Brazilian berries – that is, initial and bioaccessible values, are shown in Table 3. According to the Folin–Ciocâlteu test, estimated initial total phenolic contents follow the order: grumixama > barapiroca > nhamburi > black pitanga. Regarding flavonoids – the largest class of phenolic compounds, and which comprise anthocyanins – barapiroca surpasses grumixama, ahead of nhamburi and pitanga. However, pitanga has the highest representation of flavonoids in relation to the total phenolic content (53.2%). Anthocyanins represent the main flavonoid subclass in all fruits, comprising at least 51.4% (barapiroca) of total flavonoids, ranging from 351 to 555 μg C3G g^−1^ for black pitanga and nhamburi, respectively.

**Table 3:** Contents of total phenolic compounds (TPC), total flavonoid compounds (TFC), and total monomeric anthocyanin compounds (TMAC) before and after *in vitro* digestion

Compound	Black pitanga		Grumixama		Nhamburi		Barapiroca	
	Initial	Bioacessible	Initial	Bioacessible	Initial	Bioacessible	Initial	Bioacessible
TPC (μg GAE g^−1^)	687 ± 51d	1558 ± 4211B	4429 ± 267a	2096 ± 143A	1555 ± 118c	1725 ± 83B	2563 ± 37b	1716 ± 185B
TFC (μg CE g^−1^)	365 ± 12d	111 ± 56C	657 ± 47b	540 ± 54A	461 ± 19c	311 ± 14B	998 ± 11a	409 ± 56B
TMAC (μg C3G g^−1^)	352 ± 27b	201 ± 6B	399 ± 28b	141 ± 18B	555 ± 34a	348 ± 45A	513 ± 11a	152 ± 23B

Lower‐case letters in the same row indicate statistical differences according to ANOVA followed by a post hoc Tukey test for initial samples (*P* < 0.05). Upper‐case letters in the same row indicate statistical differences according to ANOVA followed by a post hoc Tukey test for bioaccessible samples. All results are expressed as fresh weight. GAE, gallic acid equivalents; CE, catechin equivalents; C3G, cyanidin‐3‐*O*‐glucoside.

*In vitro* gastrointestinal digestion altered the content of bioactive compound to a greater or lesser extent between fruits (Table 1). After *in vitro* digestion, grumixama continued to be the highest in phenolics, despite presenting only 52.5% of initial value, whereas the initial difference between the other three fruits ceases to exist after *in vitro* digestion. Significant lower values were also observed for the total flavonoid (17.7–69.4%) and anthocyanin (37.5–70.3%) contents for all fruits after *in vitro* digestion. Interestingly, despite nhamburi and barapiroca presenting similar initial anthocyanin values, the bioaccessible fraction values of these compounds were different at the end of digestion, suggesting the food matrix composition effect on BA.

**Figure 4:**
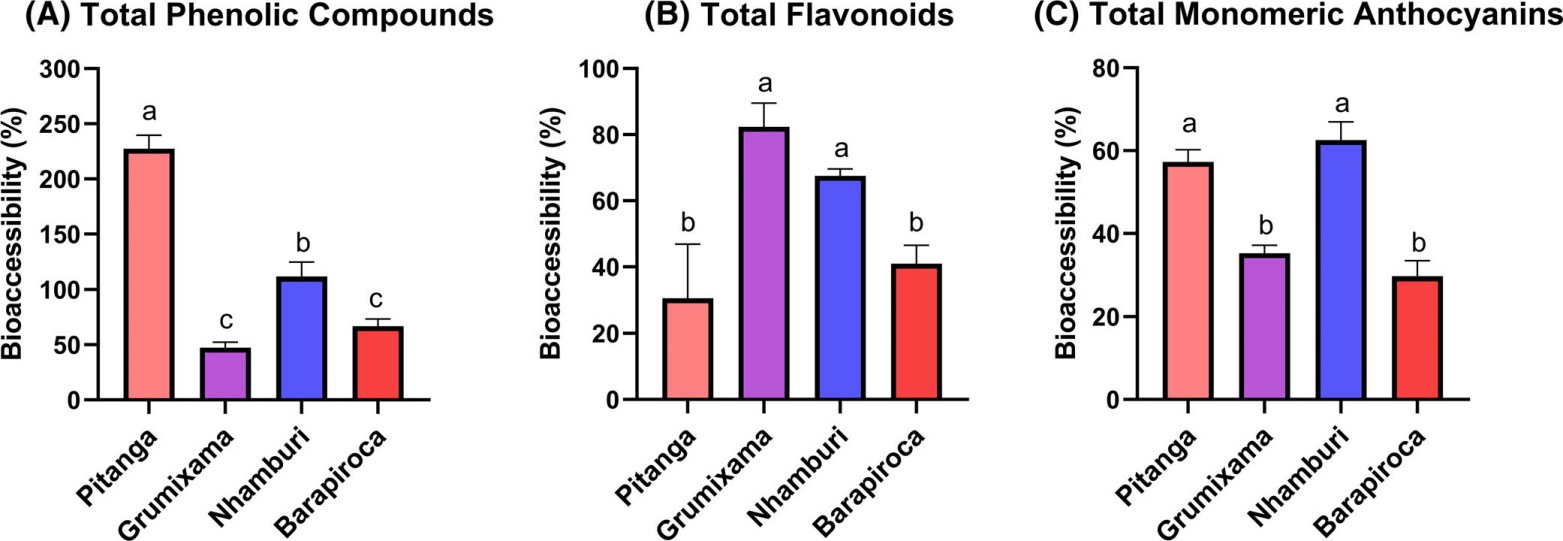
Bioaccessibility of bioactive compounds of the fruits after *in vitro* digestion. Letters above bars indicate statistical differences of bioaccessibility between fruits according to ANOVA and Tukey tests (*P* < 0.001).

**Figure 5:**
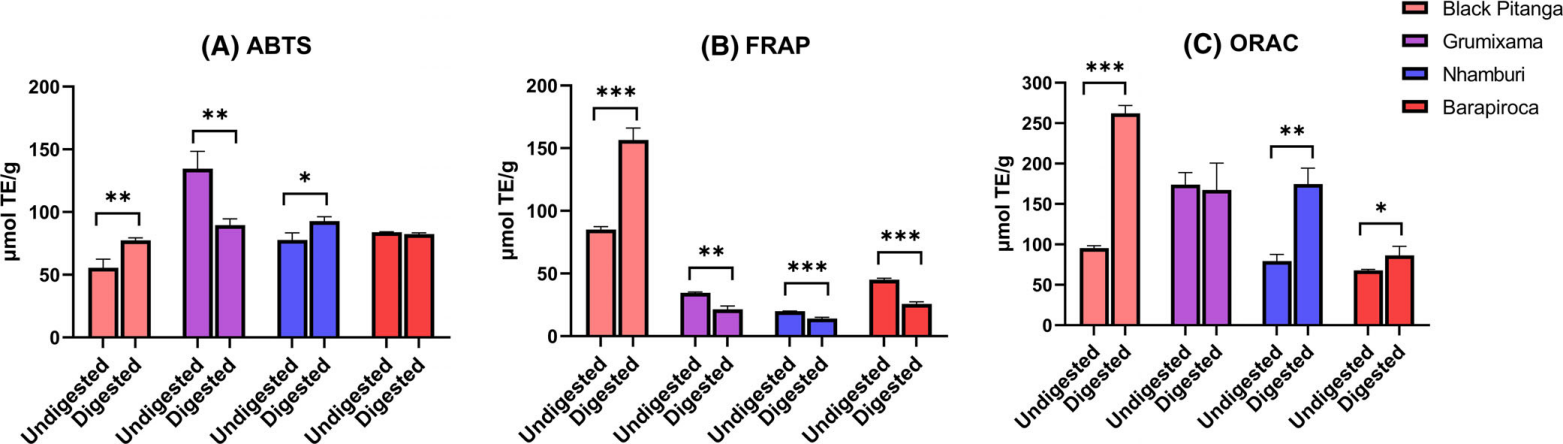
Effects of *in vitro* digestion on antioxidant activity of the fruits. Asterisks indicate statistical differences between the undigested (*ie*. initial) and digested (*ie*. bioaccessible fractions) values after *in vitro* digestion according to Student's *t*‐test (*P < 0.05; **P < 0.01; ***P < 0.001). Results are expressed as fresh weight.

BA data help predict the amount of bioactive compounds that will be available for antioxidant actions at the intestinal level. Pitanga and nhamburi presented elevated phenolic BA – over 100% – which was accompanied by the antioxidant activity results after digestion (Figs 4 and 5). Accordingly, a negative effect in antioxidant activity after digestion is observed for grumixama, related to low BA of total phenolics.

After digestion, flavonoid bioacessibility of grumixama (82.3%) and nhamburi (67.5%) was higher than pitanga (30.6%) and barapiroca (41.0%) (Fig. 4). Regarding anthocyanins, nhamburi (62.5%) and pitanga (57.3%) presented higher BA than grumixama (35.2%) and barapiroca (29.7%) (Fig. 4). Therefore, flavonoids are now represented exclusively by anthocyanins in pitanga and nhamburi bioacessible fractions, while their representation in grumixama and barapiroca was reduced to 26.1% and 37.3%, respectively.

### Proximal composition

Table 4 shows the proximate composition of black pitanga, grumixama, nhamburi and barapiroca. Overall, berries from the *Eugenia* genus (black pitanga, grumixama and barapiroca) showed lipid results without significant differences. Nhamburi was the berry with the highest lipid content in its composition. Grumixama was the fruit presenting the lowest protein content in its composition, while black pitanga, nhamburi, and barapiroca berries were similar. A lower content of total carbohydrates and total energy value was observed for grumixama. The highest content for these data was found in nhamburi.

**Table 4:** Composition of Brazilian fruits food matrix

Proximate composition (g 100 g^−1^)	Black pitanga	Grumixama	Nhamburi	Barapiroca
Moisture	85.79 ± 0.36b	88.11 ± 1.35a	80.78 ± 0.68c	85.75 ± 0.46b
Ash	0.41 ± 0.07a	0.52 ± 0.47a	0.37 ± 0.03a	0.43 ± 0.01a
Total carbohydrates	11.71 ± 0.25b	9.58 ± 1.02c	15.82 ± 0.94a	12.04 ± 0.50b
Total sugars	9.30 ± 0.23a	6.63 ± 0.89b	10.42 ± 0.70a	9.19 ± 0.35a
Total fiber	2.41 ± 0.09c	2.95 ± 0.13b	5.40 ± 0.25a	2.84 ± 0.16bc
Soluble fiber	0.79 ± 0.02ab	0.87 ± 0.03a	0.74 ± 0.04b	0.76 ± 0.03b
Insoluble fiber	1.62 ± 0.12c	2.08 ± 0.10b	4.66 ± 0.21a	2.08 ± 0.13b
Protein	1.48 ± 0.10ab	0.74 ± 0.13b	1.70 ± 0.63a	1.14 ± 0.03ab
Lipids	0.61 ± 0.28b	0.39 ± 0.13b	1.34 ± 0.21a	0.63 ± 0.09b
Energy value (kcal 100 g^−1^)	58.25 ± 3.06b	44.77 ± 2.84c	82.11 ± 3.21a	58.40 ± 1.68b

*Note*: Values are expressed as mean ± SEM (*n* = 3). Different lower‐case letters in the same row indicate significant differences among fruits for the same parameter, according to Tukey's test (*P* < 0.05).

Nhamburi was the fruit with the highest fiber content, presenting a higher content of insoluble fiber, which can be explained by the presence of seeds attached to the pulp – a characteristic of fruits of the *Rubus* genus. For grumixama and barapiroca the results of fiber content showed no significant differences. Dietary fiber results can also be compared between barapiroca and black pitanga; despite presenting a significant difference with regard to grumixama, data remained close, highlighting the similarity of the fruits belonging to the *Eugenia* genus.

Black pitanga and barapiroca are the fruits with the most similar composition, showing no statistical differences in their content of total carbohydrates, total sugar, total fiber, protein, and lipids. There was no statistical difference in ash content for all fruits. The results of the proximal composition of the fruits studied were similar to those of commercial fruits, like cherry and blueberry.

### PCA and Pearson's R correlations

PCA and Pearson's R correlation were performed in order to summarize the effect of proximal composition (Energy value, protein, carbohydrates, sugars, total fiber, insoluble fiber, soluble fiber, lipids, and ashes) and antioxidant capacity (ORAC, FRAP and 2,2′‐azino‐bis(3‐ethylbenzothiazoline‐6‐sulfonic acid (ABTS)) in the studied Brazilian berries, with the BA of total phenolic compounds (TPC), total monomeric anthocyanin compounds (TMAC), total flavonoid compounds (TFC), malvidin 3,5‐diglucoside, delphinidin‐3‐glucoside, cyanidin‐3‐glucoside, and pelargonidin‐3‐glucoside, besides the anthocyanins' stability after *in vitro* digestion. Therefore, Pearson correlation results were mostly used as a tool to support data visualization and exploratory insights. Multivariate PCA was performed to reduce the dimensionality of the data, highlight clustering patterns, and identify the most influential variables contributing to overall variability.

**Figure 6:**
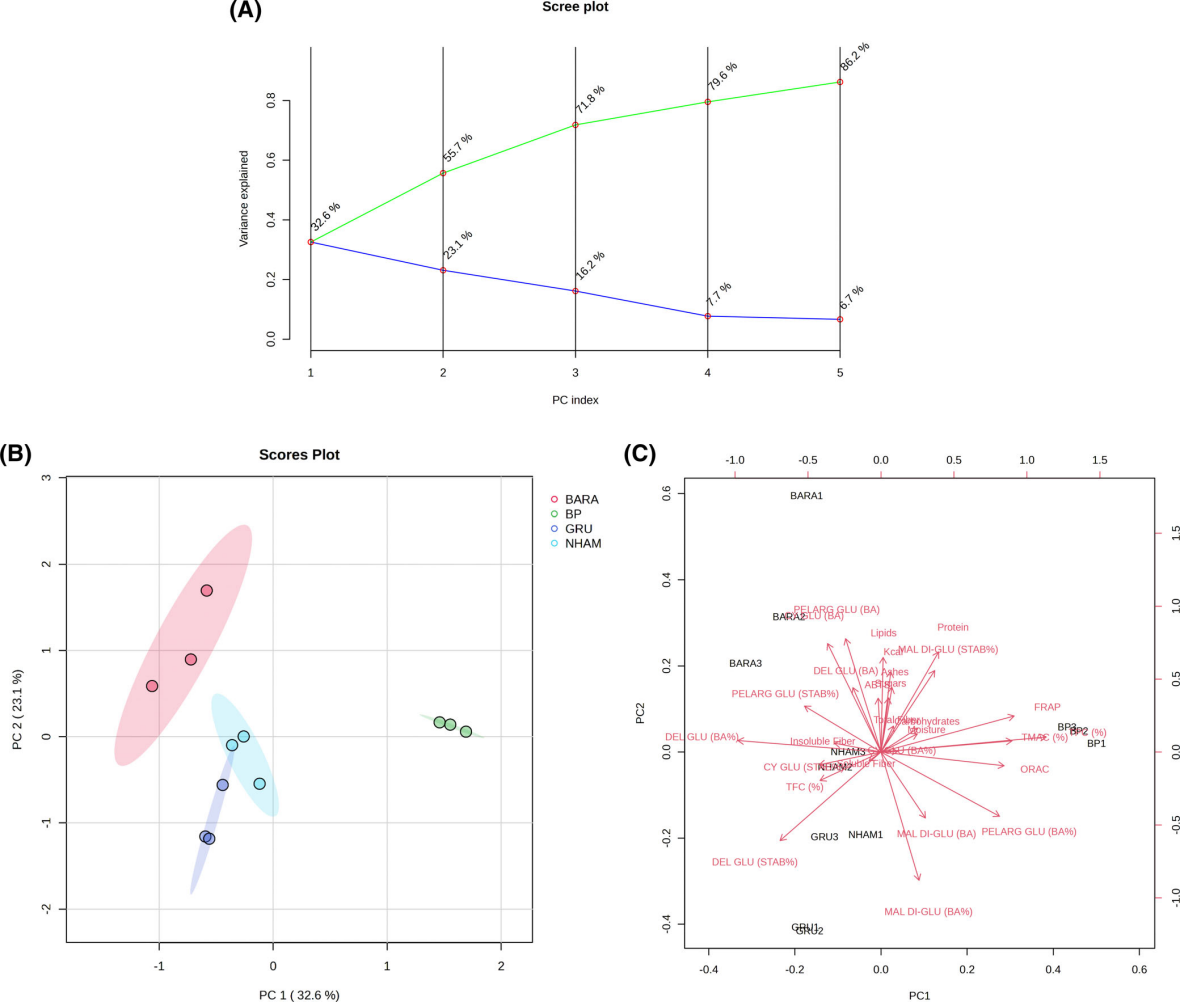
Principal component analysis of centesimal composition, antioxidant capacity, anthocyanin bioaccessibility and stability parameters of black pitanga, grumixama, nhamburi and barapiroca. (A) Scree plot of principal components. (B) Scores plot (PC1 × PC2), evidencing fruit sample clustering. (C) Biplot of samples and features. ABTS, 2,2′‐azino‐bis(3‐ethylbenzothiazoline‐6‐sulfonic acid; BA, bioaccessibility; BARA, barapiroca; CY GLU, cyanidin‐3‐glucoside; DEL GLU, delphinidin‐3‐glucoside; FRAP, ferric reducing antioxidant potential; GRU, grumixama; MAL DI‐GLU, malvidin‐3,5‐diglucoside; NHAM, nhamburi; ORAC, oxygen reactive antioxidant capacity; PELARG GLU, pelargonidin‐3‐glucoside; BP, black pitanga; STAB, stability; TFC, total flavonoid content; TMAC, total monomeric anthocyanins content; TPC, total phenolic content.

PCA components (five PCs) explained almost 86.2% of the total variation of the data (Fig. 6(A)), 32.6% being attributed to PC1 and 23.1% to PC2. As can be seen, the graph of scores (Fig. 6(B); (PC1 × PC2)) provided the two‐dimensional classification of four groups corresponding to the different fruits evaluated: black pitanga, grumixama, nhamburi, and barapiroca.

Regarding the distribution of parameters, PC1 was mostly influenced by TPC (loading: 0.41884), delphinidin‐3‐glucoside BA (%) (loading: −0.36278), FRAP (loading: 0.3374), TMAC (loading: 0.33199), ORAC (loading: 0.31194), and pelargonidin‐3‐glucoside BA (%) (loading: 0.30031). On the other hand, malvidin‐3,5‐diglucoside BA (%) (loading: −0.38615) and pelargonidin‐3‐glucoside BA (loading: 0.334108) were responsible for PC2 separation.

**Figure 7:**
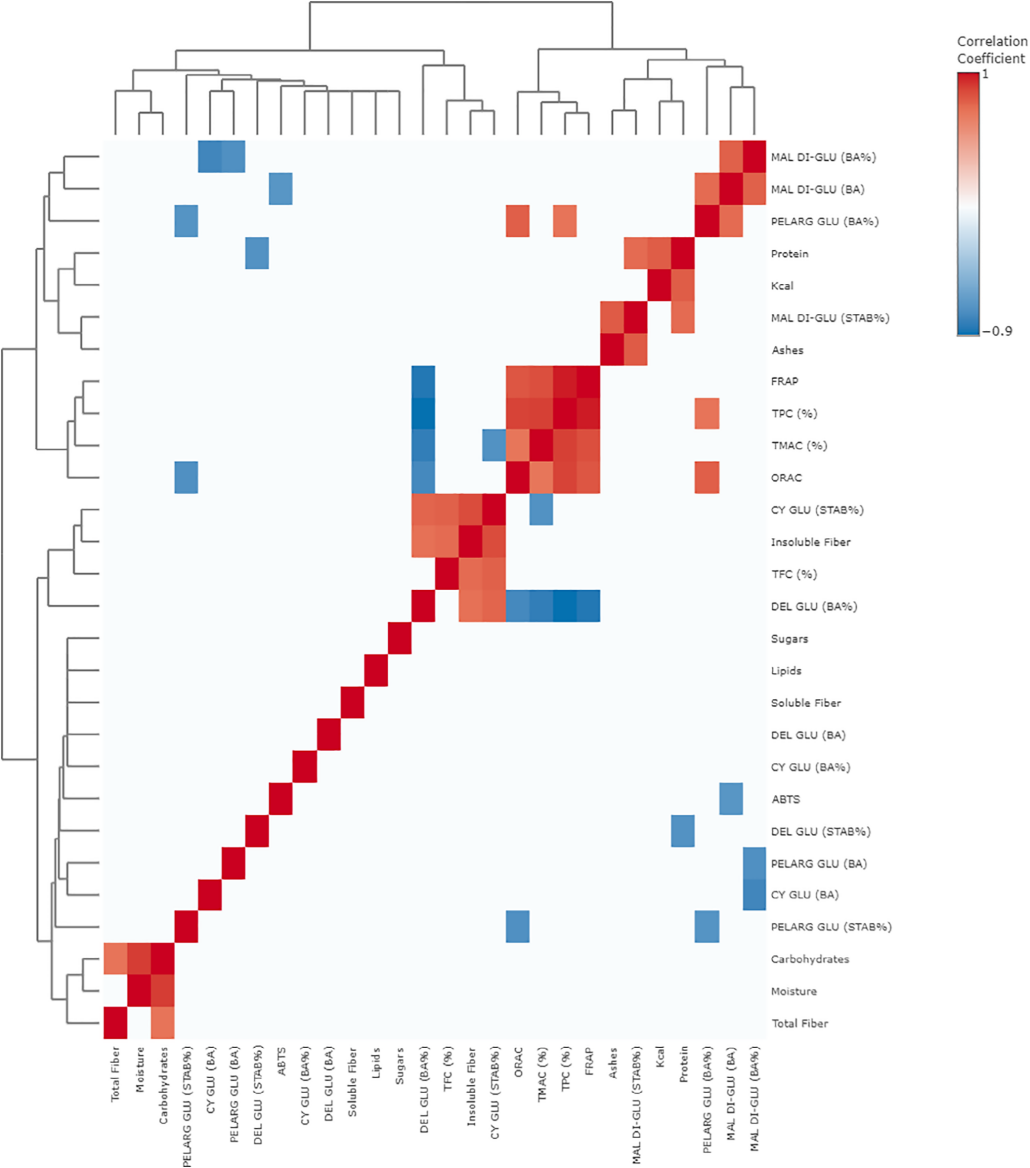
Heatmap of features of Pearson's R correlation (*P* > 0.7 cutoff). ABTS, 2,2′‐azino‐bis(3‐ethylbenzothiazoline‐6‐sulfonic acid; BA, bioaccessibility; CY GLU, cyanidin‐3‐glucoside; DEL GLU, delphinidin‐3‐glucoside; FRAP, ferric reducing antioxidant potential; MAL DI‐GLU, malvidin‐3,5‐diglucoside; ORAC, oxygen reactive antioxidant capacity; PELARG GLU, pelargonidin‐3‐glucoside; STAB, stability; TFC, total flavonoid compounds; TMAC, total monomeric anthocyanin compounds; TPC, total phenolic compounds.

In the upper left quadrant of PCA biplot (Fig. 6(D)), it is possible to notice the correlation of barapiroca with some nutritional composition parameters, such as protein, lipids, ashes, and sugar contents, as well as the absolute BA of pelargonidin‐3‐glucoside, cyanidin‐3‐glucoside, and delphinidin glucoside; whereas, on the central left, nhamburi samples were directed to the same quadrant as insoluble and soluble fiber contents, cyanidin‐3‐glucoside stability and BA (%), delphinidin‐3‐glucoside BA (%) and TFC. On the central right part of the biplot, TPC, TMAC, ORAC, FRAP, and pelargonidin‐3‐glucoside BA (%) are mostly associated with black pitanga samples. Furthermore, the lower left quadrant shows the interaction between grumixama samples with the parameters of TPC, delphinidin‐3‐glucoside stability, malvidin‐3,5‐diglucoside BA (absolute and %). Despite the clear correlations stated by the PCA biplot, no feature could be directly correlated (loading > 0.7) to PC1 or PC2. Therefore, visualization of the feature interactions was further analyzed by Pearson's R correlation (Fig. 7).

According to the heatmap of the correlation analysis, the nutritional composition of the Brazilian berries is generally more associated with the stability of anthocyanins than to its BA. Insoluble fiber has been positively correlated with cyanidin‐3‐glucoside stability (*P*: 0.851); TFC (*P*: 0.742), and delphinidin‐3‐glucoside BA (%) (*P*: 0.72). Protein (*P*: 0.743) and ash (*P*: 0.8) contents have been positively correlated with malvidin‐3,5‐diglucoside stability; whereas protein (*P*: −0.745) content has been negatively correlated with delphinidin‐3‐glucoside stability. Cyanidin‐3‐glucoside stability has been positively correlated with insoluble fiber, TFC (*P*: 0.779), and delphinidin‐3‐glucoside BA (%) (*P*: 0.764), despite the negative correlation with TMAC (*P*: −0.75). Furthermore, pelargonidin‐3‐glucoside stability only presented negative correlations, with pelargonidin‐3‐glucoside BA (%) (*P*: −0.738) and ORAC (*P*: −0.758).

In opposition, anthocyanin BA (%) is mainly connected to bioactive compound total content and antioxidant capacity parameters as follows: malvidin‐3,5‐diglucoside BA (%) has been positively correlated with its absolute BA (*P*: 0.78), and negatively correlated with pelargonidin‐3‐glucoside BA (*P*: −0.758) and cyanidin‐3‐glucoside BA (*P*: −0.816); pelargonidin‐3‐glucoside BA (%) has been negatively associated with its stability (*P*: −0.738), while malvidin‐3,5‐diglucoside BA (*P*: 0.742), TPC (*P*: 0.706), and ORAC (*P*: 0.786) have been positively correlated with this feature; delphinidin‐3‐glucoside BA (%) has been negatively associated with FRAP (*P*: −0.901), TPC (*P*: −0.945), TMAC (*P*: −0.863), and ORAC (*P*: −0.801), while being positively correlated with cyanidin‐3‐glucoside stability (*P*: 0.764) and insoluble fiber (*P*: 0.72). The absolute BA of pelargonidin‐3‐glucoside and cyanidin‐3‐glucoside have been negatively correlated with malvidin‐3,5‐diglucoside BA (%) (*P*: −0.758 and −0.816, respectively). Moreover, malvidin‐3,5‐diglucoside absolute BA has been positively associated with its BA (%) (*P*: 0.78) and pelargonidin‐3‐glucoside BA (%) (*P*: 0.742), while presenting a negative correlation with ABTS (*P*: −0.724).

## DISCUSSION

The present work points out that the chemical characterization of fruit matrices intended for human consumption is insufficient to affirm their nutritional and functional benefits. Understanding the bioavailability of bioactive compounds, like anthocyanins, is crucial for assessing their significance in human health. In this sense, exploring the behavior of bioactive compounds during oral–gastrointestinal digestion and their BA is of great relevance. Some commercial fruits have been assessed for anthocyanin BA,^35^, ^36^ while native berries of South America have been underexplored. This includes berries native to Brazil.

The basic form, 2‐phenylchromenylium, includes six main compounds: cyanidin, delphinidin, malvidin, pelargonidin, and petunidin, which are the most common anthocyanins in fruits, mostly linked to glucosides.^37^, ^38^ Currently, black pitanga displays an expected anthocyanin profile, with additional derivatives of malvidin and delphinidin that has never been identified before in other pitanga varieties. Red pitanga, for example, had its anthocyanin profile analyzed previously, and was found to contain 11 different anthocyanins, with cyanidin 3‐glucoside and delphinidin 3‐glucoside in higher abundance.^39^ In certain varieties of purple pitanga, the main anthocyanins are cyanidin 3‐galactoside and pelargonidin 3‐galactoside.^39^ Like other species within the *Eugenia* genus, black pitanga and barapiroca contain cyanidin 3‐glucoside as the primary anthocyanin. Petunidin and malvidin derivatives were identified in very low abundance in the berries analyzed, while delphinidin 3‐glucoside or its derivatives were consistently present across all berries, with barapiroca containing the highest abundance (7.5%). Commercial fruits with dark blue or nearly black colors typically exhibit a high prevalence of delphinidins, such as blackcurrants (66.7%) and blueberries (57.6%).^29^, ^30^ Nhamburi, a lesser‐known berry, has had limited scientific studies; we present for the first time the anthocyanin composition of nhamburi. The results indicate that nhamburi has an anthocyanin profile similar to raspberry, also from the *Rubus* genus: cyanidin 3‐glucoside > delphinidin 3‐glucoside > pelargonidin 3‐glucoside.^40^

The stability of anthocyanins varied among berries depending on their composition. Anthocyanins are generally unstable compounds, influenced by factors such as pH, oxygen, chemical structure, and enzyme presence. Evidence shows that anthocyanins undergo degradation during intestinal digestion, transitioning from flavylium cations in acidic conditions to colorless chalcone aglycones and conjugated metabolites in alkaline conditions.^11^ Despite this, more than 40% of anthocyanins remained stable after *in vitro* digestion across all berries analyzed.

The stability of the total monomeric anthocyanins of ripe grumixama revealed that 71% remained after *in vitro* gastric digestion, and only 4% remained after the intestinal phase. While ripe grumixama presented 4% stability, midi‐ripe grumixama presented 6%. Despite the elevated transformation of anthocyanins due to pH in the intestinal phase being demonstrated, these authors used only unspecific spectrophotometric analysis and there was no separation of the bioaccessible fraction.^41^ We also demonstrated the low stability of anthocyanins through *in vitro* digestion methods, and the effect of the fruit matrix. INFOGEST static *in vitro* digestion validates its protocol with fixed pH for oral (pH 7), gastric (pH 3) and small intestine (pH 7) digestion phases. Differences in buffering capacity of fruits were also observed, in particular for black pitanga; further studies with semi‐dynamic models of *in vitro* digestion should be considered to better understand the food matrix buffering capacity effect over stability and BA of anthocyanins. Moreover, the sugar portion linked to anthocyanins is essential for their stability. Upon release from cellular structures by oral phase amylases, anthocyanins undergo spontaneous cleavage of aglycones, producing byproducts such as protocatechuic, caffeic, gallic, and vanillic acids. Since anthocyanins have low bioavailability post‐gastrointestinal digestion, these metabolites can provide beneficial effects upon absorption or interaction with the microbiota.^42^, ^43^

Recently, the interaction of anthocyanins and mucin proteins present in saliva has been demonstrated; for example, in different profiles of wine phenolics, malvidin 3‐*O*‐glucoside showed stronger interaction with high‐molecular‐weight salivary proteins, such as mucins, than catechin, epicatechin and quercetin 3‐β‐glucopyranoside, with consequent involvement of oral astringency of wines.^44^ Detailed molecular interactions of cyanidin‐3‐glucoside and its oxidized form (cyanidin‐3‐glucoside quinone) with oral mucin have been characterized,^45^ proving that they impair oral lubrication and develop oral astringency. Cyanidin‐3‐glucoside quinone can interact covalently with cysteine of mucin. Therefore the role of salivary proteins over anthocyanin BA must be better understood in further research, helping food science and technology to improve anthocyanin‐rich functional products.

Gastrointestinal pH plays a decisive role in the stability, release, and BA of anthocyanins. Under highly acidic conditions (pH 1–3), anthocyanins remain predominantly as flavylium cations – the most stable and soluble form. As pH increases,^3^, ^4^, ^5^ deprotonation occurs, leading to the formation of pseudobases and chalcones, which are less stable, less soluble, and more prone to degradation. These structural transformations reduce their stability, influence their antioxidant capacity, and can modulate intestinal absorption. Food composition can raise or buffer gastric pH, modulating anthocyanin conversion into neutral or anionic forms.^46^

Strong evidence shows that anthocyanins are transported through gastric tissue by means of active transport mechanisms.^47^, ^48^, ^49^, ^50^ NCI‐N87 gastric cells showed significant impact on anthocyanin transport and uptake due to gastric residence time, pH, and concentration of chokeberry anthocyanins. The flavylium cations were preferentially taken up and transported, while the chalcone forms were less transported by these cell lines.^48^ Additionally, the MKN‐28 gastric cell model showed jabuticaba (*Myrciaria jabuticaba*), jambo (*Syzygium malaccense*), and jamelão (*Syzygium cumini*) anthocyanin transport, respectively of 19.7%, 9.7%, and 14.1%, while the Caco‐2 intestinal cell model resulted in 0.8%, 0.2%, and 0.3%, respectively.^50^ Therefore, stomach digestion, release, stability, BA, and absorption of berry anthocyanins are key to elucidating their antioxidant effects and potential health benefits.

In the present work we assessed the BA – that is, the anthocyanins that were released and remained soluble ready for absorption – by very sensitive and accurate mass spectrometric analysis. Our results show that the variation in anthocyanin BA levels among berries suggests that the food matrix plays a crucial role in modulating anthocyanins during *in vitro* digestion. Nhamburi and grumixama exhibited the highest bioaccessibilities observed, for cyanidin‐3‐glucoside and malvidin‐3,5‐diglucoside, indicating a relationship between fruit composition, stability during digestion, and final BA. Previous studies show that the plant cell walls, after being broken during digestion, can interact with polyphenols, resulting in a modulation of their BA.^7^ Proteins from the food matrix can influence polyphenols similarly, increasing or decreasing their BA.^10^

The TPC behavior during *in vitro* digestion found for black pitanga was similar to that previously reported for raspberry^51^ and *Prinsepia utilis* R. fruits.^52^ This suggests the release of phenolic ring molecules from polymerized polyphenols, proteins, sugars, and other macromolecules during digestive hydrolysis (acid, alkaline, or enzymatic). These results could even explain the consistently higher antioxidant activity (ABTS, FRAP, and ORAC) of black pitanga after *in vitro* digestion. Polyphenols range from simple structures such as phenolic acids to complex polymeric forms such as tannins and flavonoids.^9^ Moreover, in BA studies, the extensive handling of samples, pH conditions, or solvents applied in the extraction methods directly affect the phenolics quantification, then affecting BA interpretation.^53^ At the same time, the Folin–Ciocâlteu method could overestimate phenolic compound content due to the contribution of interferents like organic acids, reducing sugars, and amino acids, which also may be released during digestion.^54^ Although it is widely used in BA studies, this analytical limitation necessitates caution in inferences and affirms the need for more accurate methodologies for phenolic compound analysis.

The observed BA of total polyphenols exceeding 100% in black pitanga may be attributed to its elevated concentration of proanthocyanidins and hydrolysable tannins.^55^, ^56^ The hydrolysis of these polymers enhances their reactivity with the Folin–Ciocâlteu reagent. In their native polymeric state, tannins exhibit limited reducing potential because many phenolic groups are sterically hindered or engaged in intra‐ and intermolecular associations. Following hydrolysis, due to gastrointestinal digestion, tannins are broken down into low‐molecular‐weight compounds such as gallic acid, catechin, and epicatechin, which contain phenolic hydroxyl groups that are more accessible and possess greater electron‐donating capacity.^57^, ^58^

Higher antioxidant activity after *in vitro* digestion was also observed for nhamburi and barapiroca, although unaccompanied by TPC. By the same principle of the Folin–Ciocâlteu method, antioxidant properties could be attributed to protein structural alterations. During digestion, the tertiary structure of proteins can be weakened, exposing functional groups; the hydrolysis of peptide and C—H bonds can generate bioactive peptides, and the active hydrogen content can be increased, which are favorable conditions for the improvement of its antioxidant capacity.^59^ Further validation of this hypothesis through proteomic analyses or targeted biopeptide assays is necessary to confirm the presence and identity of such antioxidant peptides. In addition, protein–polyphenol binding can be formed via noncovalent and covalent interactions in food, which could favor the intestinal BA and stability of polyphenols. In this way, novel protein–phenolic compound conjugates have been identified as a promising approach for enhancing antioxidant activity nutraceutical and food products.^60^, ^61^ Importantly, both phenolic and non‐phenolic antioxidant compounds are combined to exert relevant health effects at gut level or after absorption. Furthermore, it is important to consider that lipophilic antioxidant compounds, such as carotenoids, are neglected in these polar attribute analyses, contributing to sub‐estimated antioxidant activities of the fruits before and after digestion. The applied assays used in this study focused on hydrophilic extracts, thus excluding the contribution of lipophilic antioxidant compounds. Future research should adopt integrated analytical approaches that include lipophilic fractions, such as lipophilic ORAC assays, along with chromatographic techniques such as HPLC–diode array detection or LC‐MS/MS for carotenoids and other fat‐soluble antioxidants.

A similar behavior of antioxidant activity – that is, higher values after digestion process – was reported previously for passion fruit peel and brown rice.^62^, ^63^ Conversely, in the present work, grumixama showed a different behavior from these: lower antioxidant capacity accompanied by the phenolic content after *in vitro* digestion. In agreement, significant lower antioxidant values were observed for pomegranate juice, strawberry extract, and fresh apples during digestion.^64^, ^65^, ^66^ Chen *et al*.^67^ found that 26 fruits out of 33 tested showed an improvement in ABTS antioxidant capacity after *in vitro* digestion, while only eight and one fruit increased its antioxidant capacity for FRAP and DPPH, respectively. Additionally, the authors found a strong correlation between antioxidant activity and TPC results before and after digestion. In contrast, Li *et al* ^59^ showed that ABTS and FRAP values of phenolic acids and flavonols decreased after *in vitro* digestion, while the DPPH, ABTS, and FRAP values of most flavonoids increased. Moreover, increased ORAC values were found in most phenolic acids, flavonols, and flavonoids evaluated. Therefore, the contrasting results for different fruits or fruit bioactives reinforce the complexity of antioxidant activity of bioaccessible fractions, which is beyond the phenolic compound content, profile, and the assay principle.

The remarkable biological activity of red–purple fruits is primarily associated with high concentrations of anthocyanins. The primary mechanism of action of anthocyanins is their strong antioxidant capacity. Their biological functions stem from their molecular structure, which includes two aromatic rings (A and B) and a six‐membered oxygenated heterocycle (C). It has already been proven that cyanidin has high antioxidant capacity due to its ability to absorb oxygen radicals. Moreover, delphinidin‐3‐glucoside and petunidin 3‐glucoside are capable of inhibiting NF‐κB activities through the mitogen‐activated protein kinase (MAPK) pathways, which are involved in several disease conditions. Malvidin 3‐glucoside can inhibit inflammatory responses induced by tumor necrosis factor‐α, significantly reducing the expression of pro‐inflammatory genes in *in vitro* analysis. The main mechanism of intra‐ and intercellular actions of anthocyanins are derived from their potent antioxidant capacity.^5^, ^68^, ^69^

In the present study, anthocyanin BA varied between 29.7% and 62.5% after *in vitro* digestion. A similar average of anthocyanin BA found in our study was reported for typical commercial berry fruits, such as strawberry (27%), raspberry (30%), and blueberry (36%). Scrob *et al*.^12^ were also able to evaluate the gastrointestinal digestion impact on antioxidant activity of anthocyanins and ascorbic acid from lingonberry jams through *in vitro* digestion. The results also showed that the food matrix can play an important role in the stability of bioactive compounds during digestion. As demonstrated here, different levels of BA of anthocyanins reflect a protective effect of specific food matrix components of each fruit. Soluble or insoluble fibers, proteins, and lipid constituents may interact physically and chemically with anthocyanins, controlling their release and increasing their stability during the digestion process.^70^ Furthermore, it is suggested that polyphenols themselves may enhance the BA of other antioxidant compounds, protecting them during digestion due to their radical scavenging properties.^71^ Other studies showed the direct influence of the anthocyanin profile on BA, whose lower values are mainly due to the greater capacity of being oxidized inherent to their chemical structures. Although comprehensive studies on Brazilian berries composition are limited, pitanga, grumixama, nhamburi, and barapiroca provide other food matrix compounds, like vitamin C, tannins, tocopherols, carotenoids, and minerals, which can play an important role in preserving stability and modulating BA of anthocyanins, and affecting the antioxidant activity of bioaccessible fractions.^13^, ^19^, ^72^

The proximate composition values for the berries analyzed is consistent with that expected for fruits of *Eugenia* and *Rubus* species. Grumixama presented the lowest caloric value among all berries analyzed, due to its low macronutrient content (carbohydrate, lipid, and protein), demonstrating it to be a fruit rich in water in its composition, proven through its high moisture content. Nonetheless, its results were close to those found for blueberries from different countries, such as Germany, Estonia, and Finland, demonstrating that it has nutritional potential as a berry already popularly known and commercialized.^73^ In fact, the results of the proximal composition of the fruits studied were similar to those for commercial fruits, such as cherry and blueberry.

The berries analyzed proved to be good sources of dietary fiber, especially nhamburi, as it has small seeds in its pulp, which results in an increase in the amount of insoluble fiber. This also contributed to the berry having the lowest moisture content among all those examined. Fruits of the *Rubus* genus are known for having low energy value and being composed mainly of carbohydrates and dietary fiber. The results obtained for nhamburi in both compounds were close to raspberries (*Rubus idaeus*), a commercial and popular fruit.^74^, ^75^, ^76^ The results for dietary fiber from the *Eugenia* genus were close to the composition found for cherry (2.10 g 100 g^−1^) and blueberry (2.40 g 100 g^−1^). The same could be observed for the protein content for cherry (1.06 g 100 g^−1^) and blueberry (0.74 g 100 g^−1^).^77^

Considering the food matrix effects in the digestion process, we found a correlation between centesimal composition and anthocyanins stability. Moreover, low correlation among anthocyanin BA and the macronutrients present in the Brazilian berries studied was intriguing. Only delphinidin‐3‐glucoside BA presented a positive correlation with insoluble fiber content. Among these associations, we highlight the negative correlation of delphinidin‐3‐glucoside stability with protein, while cyanidin‐3‐glucoside stability has been positively correlated with insoluble fiber content. Insoluble fiber may have been capable of preserving cyanidin‐3‐glucoside structure during simulated digestion, being detectable in the stable fraction of the compound, since most of the fiber action would occur in the non‐accessible fraction. This interference may have been detected only for cyanidin‐3‐glucoside stability, since it is present in more significant levels in the samples than the other anthocyanins evaluated.

Furthermore, the opposite correlations of some anthocyanin BA and antioxidant parameters should be highlighted. Pelargonidin‐3‐glucoside BA has been positively associated with total polyphenols, while delphinidin‐3‐glucoside BA was negatively associated. These opposite results have also been observed for ORAC features. Despite the negative correlations of delphinidin‐3‐glucoside and malvidin‐3,5‐diglucoside bioaccessibilities with ORAC, this event may be explained by some hypotheses: (i) these anthocyanins do not present the same antioxidant capacity as other original phenolic compounds that have been degraded during *in vitro* digestion, thus reducing ORAC values after the experiment; (ii) malvidin‐3,5‐diglucoside and delphinidin‐3‐glucoside physicochemical catabolites, such as phenolic acids, could exert a higher antioxidant capacity than its original compounds, which connects high BA with lower antioxidant capacity; among others.

## CONCLUSIONS

By evaluating Brazilian native berries, this study underscores the importance of considering BA when exploring their functional potential. The novelty of our work resides in providing the first comprehensive data of anthocyanin BA and stability in nhamburi, black pitanga, grumixama, and barapiroca. Traditional berries, often inaccessible to many, especially those vulnerable to NCDs, prompt the exploration of nearby, affordable, native alternatives with substantial health benefits. In this sense, the demonstration that there are Brazilian native berries with great potential to reach food markets, and present rich contents of bioaccessible bioactive anthocyanins, like the four presented here, is of utmost relevance. Black pitanga and nhamburi – overlooked fruits thriving in tropical climates – emerge as promising sources of anthocyanins since they presented higher amounts of bioaccessible anthocyanins after *in vitro* digestion. Our findings align with existing literature on anthocyanin BA, shedding light on the intricate interactions between food matrices and gastrointestinal behavior. To the best of our efforts, we tried to explain differences between fruits with regard to anthocyanin stability, BA, and antioxidant effect behavior based on their food matrix composition analysis, which proved to be essential, despite not being completely clarified. Further studies may focus on the role of minor components – that is, vitamins, lipids, and minerals. Thus, our work underscores the importance of assessing bioactive compound BA on a plant‐by‐plant basis, eschewing indirect associations or erroneous assumptions. Furthermore, our research contributes to ongoing efforts to leverage biodiversity for nutritional and commercial gains. By providing scientific support for the market potential of native fruits, our findings hold promise for enhancing consumer well‐being while safeguarding natural resources.

## Supporting information


**Data S1.** Calibration curve's information.

## Data Availability

The data that support the findings of this study are available from the corresponding author upon reasonable request.
