# Supplementary material for: Bioaccessibility of anthocyanins and bioactive compounds from Brazilian berries and their food matrix interaction: an in vitro gastrointestinal digestion study coupled to UHPLC‐ESI‐TQD‐MS/MS analysis
Source: J Sci Food Agric. 2026 Jan 29;106(6):3462–77. doi: 10.1002/jsfa.70436 (PMC12988717; doi:10.1002/jsfa.70436)
Supplement: Supplementary file 1 — Data S1. Calibration curve's information. [file JSFA-106-3462-s001.docx]

Supplementary Material (SM1) – Calibration curve’s information

| **Concentration Range** | **R2 Values** | **Line equation** |
| --- | --- | --- |
|  | **Delphinidin** |  |
| **0.18 – 0.9** | 0.9969 | y = 4E – 05x + 0.1584 |
| **1.17 – 8.5** | 0.9983 | y = 4E – 05x + 1,3793 |
| **1.7 – 34.0** | 0.9962 | y = 0.0011x + 1.8006 |
| **0.17 – 34.0** | 0.9823 | y = 2E – 05x – 1.4654 |
|  | **Cyanidin** |  |
| **0.185 – 0.925** | 0.9996 | y = 2E – 05x + 0.1626 |
| **1.85 – 9.25** | 0.9987 | y = 2E – 05x + 1.6432 |
| **0.185 – 37.0** | 0.9968 | y = 0.0006x + 0.0847 |
| **1.85 – 185.0** | 0.9991 | y = 8E – 06x – 3.5069 |
|  | **Pelargonidin** |  |
| **0.17 – 0.85** | 0.9974 | y = 2E – 05x + 0.1394 |
| **1.8 – 9.0** | 0.9973 | y = 2E – 05x + 1.5477 |
| **1.7 – 34.0** | 0.9952 | y = 0.0004 + 0.1255 |
| **0.17 – 34.0** | 0.9966 | y = 4E – 06x + 0.1403 |
|  | **Malvidin** |  |
| **0.047 – 2.35** | 0.9964 | y = 1E – 05x + 0.113 |
| **0.0235 – 0.141** | 0.9573 | y = 4E – 05x + 0.0269 |
